# Conceptual Referents, Personality Traits and Income-Happiness Relationship: An Empirical Investigation

**DOI:** 10.5964/ejop.v13i4.1394

**Published:** 2017-11-30

**Authors:** Dilwar Hussain

**Affiliations:** a Indian Institute of Technology Guwahati, Guwahati, India; Webster University Geneva, Geneva, Switzerland; The Maria Grzegorzewska University, Warsaw, Poland

**Keywords:** subjective well-being, life satisfaction, conceptual referent theory, neuroticism

## Abstract

One of the ongoing debates in social indicator and subjective well-being research is concerned with the weak relationship between objective (such as income) and subjective indicators of well-being (such as life satisfaction). Empirical studies show that the relationship between subjective and the traditional objective well-being indicators is weak. This relationship is found to be very complex and far from clear. The present study tries to shed lights behind the complexity of the relationship between income and subjective well-being (SWB) by bringing into the analysis some alternative factors such as heterogeneity in the human perception and purpose of life (conceptual referent theory) and personality traits. Conceptual referent theory of happiness proposes that people differ in their conceptual referent for a happy life and this referent plays a significant role in their judgment about happiness and life satisfaction. Results of this cross-sectional survey based on 500 individuals residing in rural and urban areas indicate that the relationship between income and life satisfaction is not very strong. Furthermore, the relationship between income and life satisfaction is contingent on a person’s conceptual referent for happiness. This study suggests that income seems to have a significant influence on life satisfaction for some people (especially with outer oriented referents) and insignificant influence for other people (especially holding inner oriented referents). Additionally, neuroticism personality trait was able to further explain the relationship between income and life satisfaction. It was observed that the individuals with higher level of neuroticism tend to get a lower level of satisfaction from income rise as compared to individuals with lower level of neuroticism.

What is the impact of income on subjective well-being (SWB) or life satisfaction? This is a significant question for various stakeholders such as political parties, governments, economists and so on. There are many policy implications associated with this question. We can judge the benefits of economic progress by exploring the impact of income on SWB (Diener & Biswas-Diener, 2009). There is extensive literature on the relationship between income and well-being (Howell & Howell, 2008). However, the relationship is very complex and far from clear. Economists are generally convinced that income is a sufficient indicator of human well-being and increasing the level of income will upsurge human well-being and life satisfaction (Lakshmanasamy, 2010). However, empirical studies show that the relationship between subjective (such as life satisfaction) and the traditional objective well-being indicators (such as income, consumption) is weak. Cross-sectional survey data show significant positive but small coefficient (Blanchflower & Oswald, 2004). For example, Diener and Oishi (2000) in a cross-sectional survey of a large sample from 19 countries reported a correlation between income and subjective well-being ranging from .02 to .38. These findings generally indicate that subjective well-being increases with income at a decreasing marginal rate. Furthermore, other evidences suggest that the link between income and life satisfaction could be more complex than that. For example, Easterlin (1974, 1995) reported no significant link between income and happiness both across countries and within individual countries. Similar findings were also reported by psychologists Diener et al. (1995) and political scientists (Inglehart, 1990). In this direction, (Diener & Biswas-Diener, 2009) after reviewing various findings related to income and subjective well-being concluded the following:

1. There are large correlations between the wealth of nations and mean reports of SWB.
2. There are mostly small correlations between income and SWB within nations (correlation appears to be larger for poor nations)
3. Economic growth in the last decades in most economically developed nations has been accompanied by a little rise in SWB
4. People who prize material goals more than other values tend to be substantially less happy

The complexity of the relationship between income and SWB is further reflected by the discrepancies in terms of objectively deprived individuals claimed to be satisfied with their life (satisfied poor) and vice versa. This phenomenon has been termed as ‘satisfaction paradox’. For example, Zapf (1984) reported two types of discrepancies between objective and subjective indicators of wellbeing. One category includes objectively well-off people but reported dissatisfaction with their life. He called them ‘frustrated privileged’. Other category included objectively deprived people but reported to be satisfied with their life. He called them ‘adapted people’. He used the term satisfaction paradox specifically for this group of people. Recently, Neff (2009) in a study with rural south Indian villages reported that about one-fifth of individuals in the lowest income quintile exhibited ‘satisfaction paradox’. He interpreted this phenomenon as a way of adaptation to poverty. Individuals learned to become satisfied with their deprived living conditions.

## Present Study

The present study tries to shed lights behind the complexity of the relationship between income and SWB by bringing into the analysis some alternative factors. Specifically, this study will investigate the following variables to explain the relationship between income and SWB-

1. Role of heterogeneity in the human perception and purpose of life (conceptual referent theory) and
2. Personality traits

These factors may provide greater insights into the dynamics of human SWB. Human beings are not just consumers of material goods. They have diverse traits and purposes in life which will influence their evaluation of life satisfaction. Therefore, money can be a good determinant of life satisfaction for some but may not be a significant factor for others. Very few studies have investigated the role of these factors in explaining the relationship between income and SWB. Although, many studies have investigated the role of personality traits in SWB, very few studies have investigated the role of personality traits in the relationship between income and SWB. This study will be a step forward in this direction.

This study will investigate following specific research questions:

1. Does income have an influence on life satisfaction or subjective well-being? Whether people in lower income tend to be less satisfied with their life as compared to people with higher income?
2. What is the relationship between objective indicator (income) and subjective indicator (life satisfaction/subjective well-being) of well-being? Is there any discrepancy between objective and subjective indicators of well-being?
3. To what extent people show satisfaction paradox? That is, do people report to be satisfied with their life despite being financially poor? If so, can income be used as a good proxy for subjective well-being?
4. How can we explain ‘satisfaction paradox’? Can individual heterogeneity (personality) and heterogeneity in the conceptual references for happiness play a significant role in satisfaction paradox?

## The Concept and Measurement of Subjective Well-Being (SWB)

SWB has been conceptualized in many ways. Veenhoven (1984) defined SWB as the degree to which an individual judge his/her overall quality of life as a whole in a favorable way. Diener (2009) reported that SWB comprises people’s long-term levels of pleasant affect, lack of unpleasant affect, and life satisfaction. Affect balance refers to pleasantness minus unpleasantness of one’s emotional life. Life satisfaction denotes to the conscious global judgment of one’s life. Diener (1984) suggested three properties of SWB. They are:

1. It is subjective and resides within the experience of an individual.
2. It is not just the absence of negative factors, but also includes positive measures.
3. It includes a global assessment rather than only a narrow assessment of one life domain.

In general, SWB refers to the well-being as declared by the person on a single or a group of questions about his/her well-being. It is a direct measure of well-being and substantially differs from the academically oriented analytical theory of knowledge. SWB implies bottom-up rather than top-down approach to understanding human well-being (Rojas, 2006). Sometimes the term ‘happiness’ is used synonymously with SWB. However, most authors avoid the term ‘happiness’ because of its varied meanings in common parlance (Diener, 2009).

SWB is commonly measured in terms of life satisfaction. Affect balance (positive and negative emotions) is also used as an additional measure of SWB. The link between the cognitive (life satisfaction) and affective (emotions) is a matter of debate and some authors questioned this dichotomy (Lyubomirsky, 2001). Life satisfaction is generally considered as close to the philosophers’ conception of wellbeing, which involves a person’s judgment of her life (Tatarkiewicz, 1976). The conception of life satisfaction may be more relevant for public policy and development strategy than a wellbeing assessment based on transitory affective states (Rojas, 2007). The conception of life satisfaction is expected to be less volatile and cognitively oriented (Sirgy et al., 1995). Therefore, this study measures SWB in terms of life satisfaction.

## The Conceptual Referent Theory of Happiness (CRT)

Rojas (2005) proposed conceptual reference theory of happiness to explain the heterogeneity of conceptual referents people have when they appraise their life or answers SWB question. This theory states that a person must have a conceptual referent for what a happy or satisfied life is and they must be using that referent to evaluate their quality of life. Therefore, a person’s judgment about his or her wellbeing is contingent on his/her conceptual referent for happiness (Rojas, 2005). This theory further stresses the heterogeneity in the conceptual referents people use while evaluating their life. This heterogeneity provides an explanation for people behaving differently in their pursuit of happiness. Rojas (2005) further states that upbringing and socio-cultural factors may influence the kind of conceptual referent a person develops to define a happy life.

To understand the heterogeneity in the conceptual referents for happiness, Rojas (2005) identified eight conceptual referents for happiness after reviewing various philosophical schools of thoughts associated with happiness. These conceptual referents include- Stoicism, Virtue, Enjoyment, Carpe Diem, Satisfaction, Utopian, Tranquility, and Fulfillment. Table 1 gives a brief description of each of these referents.

**Table 1:** Typology of Conceptual Referents for Happiness

Conceptual Referents	Brief Description	Associated Phrase	Orientation (Inner/Outer)
Stoicism	Happiness is a permanent state of contentment with life and with what happens in life. This state implies renunciation, austerity, acceptance, and resignation; taking things as they are and as they come out.	Happiness is accepting things as they are	Inner
Virtue	Happiness is a spiritual state produced by the feeling of acting properly, according to one’s consciousness	Happiness is a sense of acting properly in our relations with others and with ourselves	Inner
Enjoyment	Happiness is joyfulness and absence of pain; it is the enjoyment of those goods that provide comfort. It is the satisfaction of all human needs and wants	Happiness is to enjoy what I have got in life	Outer
Carpe diem	Happiness is the present pleasure and gratification, it is about enjoying now as much as possible	Happiness is to enjoy every moment in life	Somewhat Outer
Satisfaction	Happiness is a feeling of life’s elation that comes with an intuitive judgment about oneself and about one’s surrounding world	Happiness is being satisﬁed with what I have and what I am	Outer
Utopian	Happiness is an ideal that guides human action. It is perfection itself conceptualised as the synthesis of virtue and pleasure. It is a desired, yet unreachable good, at least in this life	Happiness is an unreachable ideal we can only try to approach	Inner
Tranquillity	Happiness is a state of tranquillity, the absence of worries that takes place with prudence, moderation, measurement, and judicious wants	Happiness is in living a tranquil life, not looking beyond what is attainable	Somewhat Inner
Fulfilment	Happiness is the realization of our nature and the fulfilment of our essence as human beings. Happiness is in that activity that constitutes the ultimate goal of each human being	Happiness is in fully exercising our capabilities	Somewhat Outer

*Note.* From Rojas (2005, 2007).

Furthermore, as indicated in Table 1, Rojas (2007) divided these conceptual referents based on their inner versus outer orientation. Inner oriented conceptual referents emphasize inner conditions as the main foundation for happy life. On the other hand, outer oriented referents emphasize that happiness emerges from a person’s relation to his or her external world. External conditions will have a greater effect on happiness for such a person. According to the author, this distinction “implies an oversimplification of the philosophical thought; however, the attempt may be useful to understand the nature of the relationship between a person’s income and her happiness appraisal” (Rojas, 2007, p. 8). Therefore, this dichotomous classification is an oversimplification and not a definitive classification. It seems to indicate that some conceptual referents are more oriented towards inner dimensions and others towards outer orientation. However, this distinction may have significant implications for investigating the relationship between income and life satisfaction. In this connection, it can be hypothesized that income will play a stronger role in a person’s happiness if he/she holds a conceptual referent with an outer orientation (Rojas, 2007).

Most studies investigating the relationship between income and happiness (particularly in economics) assumes a universal conceptual referent for happiness or well-being and makes conclusions and policies based on that assumption. Such conclusions can be erroneous (Rojas, 2005). Despite the theoretical significance of this theory, no study has investigated this theory in the context of income and happiness (except Rojas, 2007). Consequently, this study investigates CRT to better understand the relationship between income and life satisfaction.

## Income, Personality Traits, and SWB

Personality is regarded as one of the strongest predictors of subjective well-being (DeNeve & Cooper, 1998). Some authors estimate that between 44% and 52% of the variation in well-being is predicted by individual differences (Lykken & Tellegen, 1996). Interestingly, Lucus and Diener (2009) reported that the strong influence of personality on SWB is one of the most replicable findings emerged in the last decade; but, personality theory has not found a central role in well-being research. They further suggested that one of the reasons behind this paradox is that generally happiness is considered as an outcome of life conditions and from this perspective strong effect of personality and weak effect of situations seems counter-intuitive. However, this finding makes more sense when SWB is also considered as an ongoing process and not just an outcome.

Among the personality traits, extraversion has received greatest attention and a huge body of literature supported its significant association with SWB (Argyle & Lu, 1990; Rusting & Larsen, 1997). However, close examination of these studies revealed that the effect size of extraversion is quite small as compared to the effect size of neuroticism (Vittarsø, 2001). In this direction, DeNeve and Cooper (1998) in a meta-analysis found that neuroticism was the strongest predictor of both life satisfaction and happiness and extraversion contributed somewhat in explaining the variance in positive affect.

Although the influence of personality on SWB has been studied, very few studies addressed the role of personality in the relationship between income and SWB. Personality may have significant implications when studied in relation to income and SWB. It will help us to understand how economic incentives might influence individuals with different personality traits (Boyce & Wood, 2011). It is very likely that an individual’s marginal utility of income depends on their personality (Boyce & Wood, 2011). Further, the relationship between income and well-being may be dependent on an individual’s personality type (Boyce & Wood, 2011). This potential moderating effect of personality in the relationship between income and SWB has not received proper attention from researchers. Furthermore, personality could be one of the important explanatory factors that might provide insights into the phenomenon of ‘satisfaction paradox’ and a weak relationship between income and SWB. Therefore, the study investigates the moderating effect of personality traits in the relationship between income and SWB.

## Method

### Participants

500 participants (367 males and 133 females) from diverse background (200 from rural areas and 300 from urban areas) were selected randomly. Their age ranged from 19-56 with a mean of 32. Monthly household income ranged from Rupees 2,000 to 600,000 with mean value 30,000. About 70% were residing in nuclear and 30% joint family structure. Their education ranged from primary (30%), secondary (21%), higher secondary (20%), graduation (18%), and post-graduation (11%). Caste category included general (58%), Other Backward Caste (20.4%), Scheduled Caste (15.4%), and Scheduled Tribe (5.5%).

### Procedure

Participants were selected from both rural and urban areas nearby Guwahati city of Assam, India. Upon receiving their consent, participants were briefed about the purpose of the study. They were also told about the voluntary nature of participation. No specific incentives were given to the participants. After receiving their consent, a booklet comprising of all the questionnaires were handed over to them which were collected after few hours. Some participants asked for more time and returned the questionnaires after few days.

### Measures

The survey gathered information regarding the following variables:

**Demographic and Social Variables:** age, gender, background (rural/urban), caste category and education.

**Economic Variable:** Household income per month is used as the *income variable*, which means that the individual’s marginal utility will be based on changes to the income of the household that the individual is attached to.

**Subjective Wellbeing:** It is assessed in terms of *satisfaction with life* in general. The question asked was: ‘Taking everything into account, how satisfied are you with your life as-a-whole in general?’ The scale ranged from one to seven (‘very satisfied’ to ‘very dissatisfied’). Single item life satisfaction scale showed substantial validity and performed similar to multiple item satisfaction with life scale. Cheung and Lucas (2014) reported that single-item life satisfaction measures demonstrated substantial degree of criterion validity with the more psychometrically sound multi item satisfaction with life scale (SWLS) (zero-order *r* = 0.62 – 0.64; disattenuated *r* = 0.78 – 0.80). Furthermore, patterns of correlations and statistical significance with theoretically relevant variables were the same across single-item measures and the SWLS.

**Conceptual Referents for Happiness:** Once a person answered the life satisfaction question, he/she was presented with a list of phrases (presented in Table 1) and asked to select that phrase he/she related happiness/life satisfaction to. The respondent was asked to select just one phrase from the eight alternative phrases. This measurement of conceptual referents was followed from Rojas (2007).

**Personality Traits:** Big Five Inventory (BFI; John, Donahue, & Kentle, 1991) is a 44 item assessment of five personality traits: Openness, Extraversion, Agreeableness, Conscientiousness, and Neuroticism. The scale asks respondents the extent to which they agree that a particular characteristic applies to them on a 5 point Likert scale (1 = Strongly disagree to 5 = strongly agree). Reliability of this scale for this study was good. Reliability for this study was good. Alpha values are .75, .78, .73, .80, .76 for openness, extraversion, agreeableness, conscientiousness, and neuroticism respectively.

## Results

### Life Satisfaction

In the present sample, the assessment of general life satisfaction (Table 2) showed that largest numbers of respondents are dissatisfied with their lives (47.6%). On the other hand about 41.3% respondents reported to be satisfied with their lives. However, very small percentage of the respondents reported to be either ‘very satisfied’ (3.3%) or ‘very dissatisfied’ (5.6%).

**Table 2:** General Life Satisfaction

Life Satisfaction Code	Life Satisfaction Categories	%	Aggregate %
Dissatisfied			47.6
	Very dissatisfied	5.6	
	Dissatisfied	21.6	
	Rather dissatisfied	20.4	
Neutral			11.1
	Neither satisfied nor dissatisfied	11.1	
Satisfied			41.3
	Rather satisfied	19.1	
	Satisfied	18.5	
	Very satisfied	3.7	
Total		100.0	100.0

Group difference analysis showed that there is no significant difference in mean life satisfaction score of male (*M* = 3.88; *SD* = 1.70) and female (*M* = 3.85, *SD* = 1.64) sample; *t*(298) = .073, *p* = .942. However, there was a significant difference in the mean life satisfaction score of rural (*M* = 3.37, *SD* = 1.69) and urban (*M* = 4.31, *SD* = 1.54) sample; *t*(298) = 3.706, *p* < .001.

### Income, Life Satisfaction and Satisfaction Paradox

In the present sample, the correlation coefficient between income and life satisfaction is positive and statistically significant (*r* = .23, *p* = .05). However, this relationship is weak as the coefficient is small. Table 2 and 3 shows the life satisfaction level for every income quintile. Table 3 indicates that income seems to have a positive influence on life satisfaction as mean life satisfaction progressively increases from lowest (*M* = 3.30) to highest income quintile (*M* = 4.54). This finding indicates that on average people with low income are less satisfied with their lives as compared to their higher income counterparts. Furthermore, Table 3 indicates that more people located in the lower income quintile (62.5% and 63.6% in the 1^st^ and 2^nd^ income quintile respectively) reported dissatisfaction with their life as compared to higher income quintile (37.5% and 26.9% in the 4^th^ and 5^th^ income quintile respectively). Similarly, more number of people located in higher income quintile (60% and 53.8% in the 4^th^ and 5^th^ income quintile respectively) reported satisfaction with their life as compared to lower income quintile (32.5% and 27.3% in the 1^st^ and 2^nd^ income quintile respectively). Thus, money does buy happiness and satisfaction. However, income may not always determine life satisfaction as Table 3 clearly indicates the even in the lower income quintile who can be considered poor as per the conventional measure of poverty, a large percentage of people expressed high satisfaction with their lives (32.5% and 27.3% in the 1^st^ and 2^nd^ quintile respectively). Furthermore, more number of people reported satisfaction with their lives in the 4^th^ quintile (60%) as compared to 5^th^ quintile with higher income (53.8%). People expressed high life satisfaction as well as dissatisfaction with their lives at all income levels.

**Table 3:** Mean Life Satisfaction and General Life Satisfaction (in %) by Household Income Quintiles

Household Income Quintile	Dissatisfied (%)	Neutral (%)	Satisfied (%)	*M* (Life Satisfaction)	*SD*
Lowest quintile	62.5	5.0	32.5	3.30	1.87
2^nd^ quintile	63.6	9.1	27.3	3.27	1.63
3^rd^ quintile	47.1	23.5	29.4	3.74	1.35
4^th^ quintile	37.5	2.5	60.0	4.45	1.39
Highest quintile	26.9	19.2	53.8	4.54	1.49

There are about 32.5% of people in the 1^st^ quintile and 27.3% in the 2^nd^ quintile exhibited satisfaction paradox phenomenon (Table 3). They expressed satisfaction with their life despite belonging to the lowest income quintiles. To understand the possible reasons behind this phenomenon, individual heterogeneity (personality) and heterogeneity in the conceptual referents for happiness was explored. Following sections report the findings associated with these concepts.

### Conceptual Referents for Happiness

Table 4 presents the distribution of conceptual referents for happiness across the sample. This distribution clearly reveals that there is heterogeneity in the conceptual referents among people while evaluating their life satisfaction or happiness. It is possible that one of the reasons behind the weak relationship between income and subjective well-being is the heterogeneity in the conceptual reference people use to evaluate their life. Furthermore, Table 4 reveals mean life satisfaction scores of people with different conceptual referents.

**Table 4:** Sample Distribution and Mean Life Satisfaction Scores of Conceptual Referents

Conceptual Referent	Associated Phrase	Percent	Mean LS	*SD*
Stoicism	Happiness is accepting things as they are	7.4	4.58	1.505
Virtue	Happiness is a sense of acting properly in our relations with others and with ourselves	11.7	4.42	1.502
Enjoyment	Happiness is to enjoy what I have got in life	11.1	3.22	1.768
Carpe diem	Happiness is to enjoy every moment in life	22.2	3.97	1.483
Satisfaction	Happiness is being satisﬁed with what I have and what I am	15.4	3.44	1.960
Utopian	Happiness is an unreachable ideal we can only try to approach	5.6	3.89	1.364
Tranquility	Happiness is in living a tranquil life, not looking beyond what is attainable	6.8	5.09	1.868
Fulfillment	Happiness is in fully exercising our capabilities	19.8	4.03	1.694
Total		100.0	3.99	1.701

It is observed that the average life satisfaction score of the whole sample is 3.99 and average life satisfaction is little higher for people with referents such as Tranquility, Stoicism, virtue, fulfillment. However, people with conceptual referents such as Carpe diem, satisfaction, enjoyment, and utopian reported lower life satisfaction as compared to whole sample average. Therefore, it seems that some conceptual referents could be superior in terms of achieving more happy and satisfying life. However, mean difference is not large enough to make a definitive conclusion.

Further, it is possible that the difference in the average life satisfaction score is influenced by socio-demographic variables. Therefore, a regression analysis was conducted to see the effect of conceptual referents on life satisfaction after controlling socio-demographic variables (age, gender, background, family income). Table 5 shows the result of regression analysis.

**Table 5:** Conceptual Referents Predicting Life Satisfaction After Controlling Socio-Demographic Variables

Predictor	β	*R^2^*
Step 1		.123**
Age	-.097	
Gender	.088	
Background	.158	
Income	.215*	
Step 2		.248**
Stoicism	-.102	
Virtue	-.317**	
Enjoyment	-.450**	
CarpeDiem	-.536**	
Satisfaction	-.442**	
Utopian	-.215*	
Fullfillment	-.483**	

*Note.* Tranquility conceptual referent is taken as a reference in the analysis. Conceptual referent categories were coded into 7 dummy codes.

*p < .05. **p < .01.

Tranquility conceptual reference was taken as reference in the regression analysis as it had the highest average life satisfaction score. Conceptual referents significantly predicted life satisfaction after controlling demographic covariates (*R^2^* = .248, *p* < .01). Result shows that under identical socio-demographic conditions, people with tranquility reported higher life satisfaction as compared to virtue (β = -.317, *p* < .01), enjoyment (β = -.450, *p* < .01), carpe-diem (β = -.536, *p* < .01), satisfaction (β = -.442, *p* < .01), utopian (β = -.215, *p* <.05), and fulfillment (β = -.483, *p* < .01). However, there was no significant difference between the people with tranquility and stoicism (β = -.102, *p* < .418) conceptual referent. Therefore, there is some indication from the present sample that people with conceptual referents such as tranquility and stoicism declare relatively higher life satisfaction as compared to other conceptual referents under identical socio-demographic variables.

### Income, Life Satisfaction and Conceptual Referents

A series of eight separate regression analysis was conducted after clustering people based on their conceptual referents. The objective of these regression analyses was to test whether income predicts life satisfaction for people holding a particular conceptual referent. Other socio-demographic variables were controlled in each of these regression analyses. Logarithmic transformation was done to the scores of household income variable in order to reduce the skewness of data. Table 6 indicates that the relationship between income and life satisfaction is statistically significant for people holding conceptual referents such as ‘satisfaction’ (β = .501, *p* < .01) ‘enjoyment’ (β = .641, *p* < .05) and ‘fulfillment’ (β = .408, *p* < .05). Interestingly, these conceptual referents are outer-oriented. However, income is not statistically significant for people holding conceptual referents such as stoicism, virtue, carpe diem, utopian, and tranquility. Most of these referents are inner oriented. Therefore, this study suggests that income seems to have a significant influence on life satisfaction for some people (especially with outer oriented referents) and insignificant influence for other people (especially holding inner oriented referents).

**Table 6:** Relationship Between Life Satisfaction and Logarithm of Income by Conceptual Referent

Conceptual Referent	β	*SE*	*R^2^*
Stoicism	.039	.402	.002
Virtue	.165	.278	.027
Enjoyment	.641*	.389	.411**
Carpe diem	.066	.457	.004
Satisfaction	.501**	.322	.251**
Utopian	.306	.634	.094
Tranquility	.343	.296	.117
Fulfillment	.408*	.274	.167*

*p < .05. **p < .01.

### Income, Life Satisfaction, and Personality

This study also made an attempt to explore the role of personality traits in explaining the relationship between income and life satisfaction. For this purpose, the role of big five personality traits (extraversion, neuroticism, agreeableness, conscientiousness, and neuroticism) in explaining the relationship between income and life satisfaction was investigated. Only neuroticism turned out to be a significant moderator between income and life satisfaction among the big five factors. Summary of the moderated regression analysis is shown in the Table 7.

**Table 7:** Moderating Effect of Neuroticism Between Household Income and Life Satisfaction

Variables	β	*SE*	*R^2^*
Household income	.149	.127	.165**
Neuroticism	-.153	.068	
Income x Neuroticism	-.358**	.193	

*p < .05. **p < 01.

**Figure 1:**
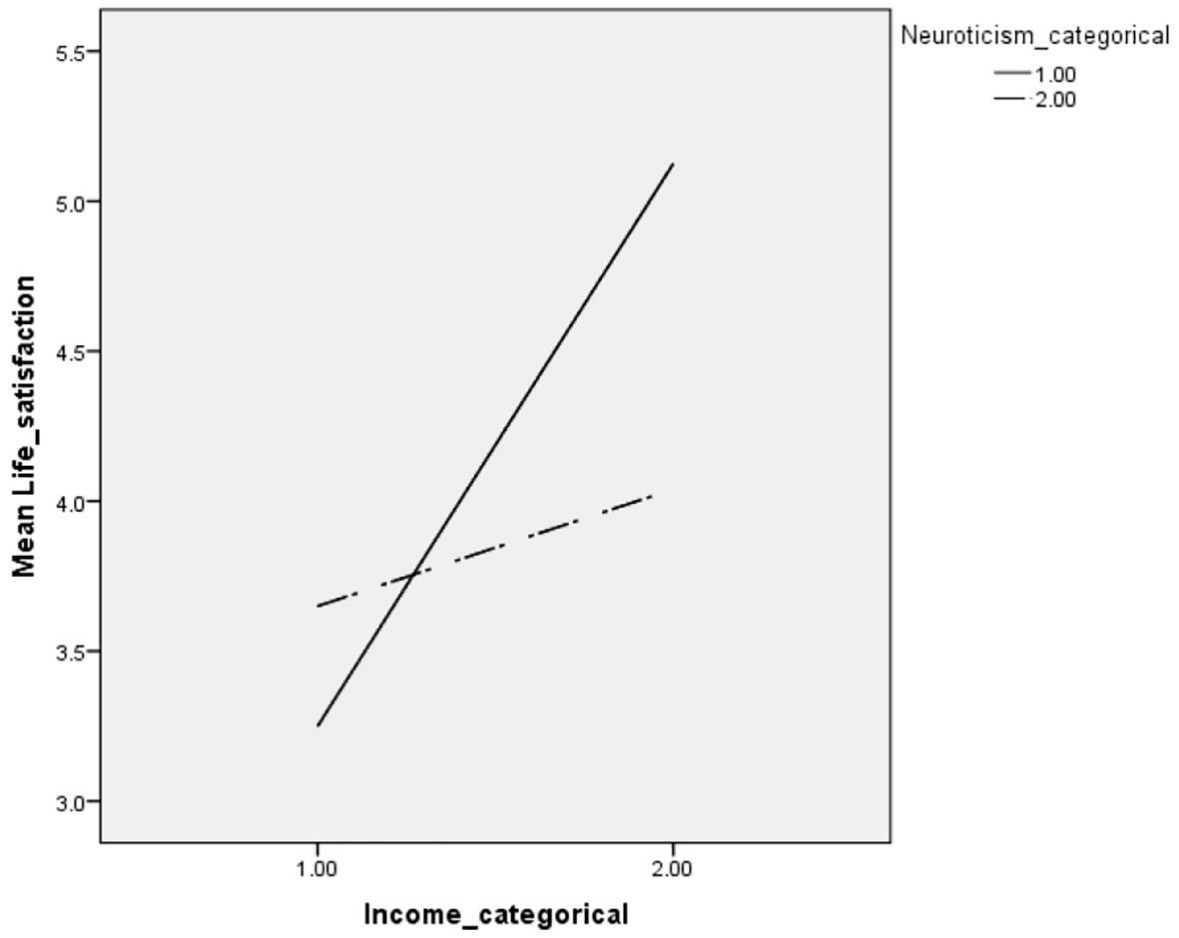
Moderating Effect of Neuroticism Between Income and Life Satisfaction

Moderated regression analysis was conducted after centering the scores of both income and neuroticism and creating an interaction term between them. Moderated regression analysis reveals a significant income personality interaction effect (β = -.358, *p* < .01). Demographic variables were used as control variables. Specific nature of moderating effect of neuroticism can be seen in the interaction graph in Figure 1 which was drawn after doing the median split of both neuroticism and household income.

It is clear from the Figure 1 that individuals with higher level of neuroticism (dashed line) tend to get a lower level of satisfaction from income rise as compared to individuals with a lower level of neuroticism. Furthermore, As compared to individuals with lower level of neuroticism, individuals with higher level of neuroticism reported more satisfaction when they are poorer but they reported less satisfaction when they are richer.

## Discussion

This study seems to indicate that income influences life satisfaction to some extent. For example, on average people with low income are found to be less satisfied with their lives as compared to their higher income counterparts. However, the correlation coefficient between income and life satisfaction is not very strong (*r* = .23). This finding is consistent with a large number of previous studies that have shown a weak relationship between income and measures of SWB. Furthermore, People expressed high life satisfaction as well as dissatisfaction with their lives at all income levels. A large percentage of participants also displayed ‘satisfaction paradox’. That is, many people with low income reported satisfaction with life and vice versa. Therefore, a rise in income may not always ensure a rise in life satisfaction, that is, many individuals may show a discrepancy between their objective and subjective indicators of well-being. These findings indicate that income by itself may not be considered a good proxy for SWB. It is not always correct to determine people’s well-being based on objective indicators such as income. The efficacy of income in generating well-being has been overestimated by Economist and other social scientists. Such assumptions should be carefully examined in the light of empirical evidence.

This investigation further explored two potential explanations for the weak relationship between income and life satisfaction: The role of heterogeneity in people’s purpose in life (conceptual referents for happiness) and heterogeneity in personality traits. The idea behind selecting these factors was that the human beings are not just consumers of commodities, but, they are more than that. They have subjective perceptions, different traits and purposes in life. Such factors will always play a critical role in determining the role of income on life satisfaction. These factors may help us to gain better insights in understanding the complex relationship between income and life satisfaction.

The study of conceptual referents for happiness clearly reveals that there is heterogeneity in the conceptual referents among people while evaluating their life satisfaction or happiness. People have different things in their mind when they evaluate their life satisfaction. This heterogeneity in conceptual referents may have significant implications for life satisfaction and its relationship with income. For example, results showed that the average life satisfaction is little higher for people with referents such as Tranquility, Stoicism, virtue, fulfillment. However, people with conceptual referents such as Carpe diem, satisfaction, enjoyment, and utopian reported lower life satisfaction as compared to whole sample average. When a regression analysis was conducted to see the effect of conceptual referents on life satisfaction after controlling socio-demographic variables, conceptual referents such as tranquility and stoicism declare relatively higher life satisfaction as compared to other conceptual referents. Therefore, there is some indication from the present sample that people with certain conceptual referents such as tranquility and stoicism could be superior in terms of achieving more happy and satisfying life.

When separate regression analysis was conducted after clustering people based on their conceptual referents, the relationship between income and life satisfaction was found to be statistically significant for people holding conceptual referents such as ‘satisfaction’ ‘enjoyment’ and ‘fulfillment’. Interestingly, these conceptual referents are outer-oriented. However, income is not statistically significant for people holding conceptual referents such as stoicism, virtue, carpe diem, utopian, and tranquility. Therefore, this study suggests that income seems to have a significant influence on life satisfaction for some people (especially with outer oriented referents) and insignificant influence for other people (especially holding inner oriented referents). It is clear that the relationship between income and life satisfaction is contingent on the conceptual referent a person holds. This finding lends support the conceptual referent theory and its predictions.

Among the big five personality traits only neuroticism turned out to be a significant moderator between the relationship between income and life satisfaction. Specific nature of the interaction effect shows that individuals with higher level of neuroticism tend to get a lower level of satisfaction from income rise as compared to individuals with a lower level of neuroticism. Furthermore, As compared to individuals with a lower level of neuroticism, individuals with a higher level of neuroticism reported more satisfaction when they are poorer but they reported less satisfaction when they are richer. Proto and Rustichini (2015) reported that neuroticism is associated with higher sensitivity to negative emotions and outcomes, threats and punishments. Therefore people with higher neuroticism trait experience higher sensitivity to losses and meeting expectations. They further suggested that neurotic people choose life scenarios with a lower level of satisfaction and expects that the cost of being rich is high for them and consequently predicts less life satisfaction. Therefore, this could be one reason why neurotic people derive less satisfaction with rise in come.

### Conclusions and Implications

This investigation finds out that the relationship between objective (such as income) and subjective indicators (such as life satisfaction) is not very strong. It has also provided support to the presence of ‘satisfaction paradox’, that is, people report to be satisfied with their life despite having very low income. It is not always correct to determine a person’s well-being only on the basis of objective indicators such as income and other socioeconomic variables. It is clear that human well-being depends on many other psychological factors beyond these objective indicators.

This study investigated two potential factors explaining satisfaction paradox and the weak relationship between income and life satisfaction: The role of heterogeneity in human personality traits and life purposes. This study lends support to the idea of heterogeneity in the conceptual referents for happiness across people. Thus, people differ in how they judge their lives to assess their well-being or life satisfaction. Furthermore, the heterogeneity of conceptual referents shed some light on the nature of the relationship between income and life satisfaction. It was observed that the relationship between income and life satisfaction is contingent on a person’s conceptual referent for happiness.

Income seems to have a significant influence on life satisfaction for some people (especially with outer oriented referents such as enjoyment and fulfillment) and insignificant influence for other people (especially holding inner oriented referents such as stoicism and virtue). This difference may be one of the potential factors explaining the weak relationship between the income and SWB.

Additionally, neuroticism personality trait was able to further explain the relationship between income and life satisfaction. It was observed that the individuals with a higher level of neuroticism tend to get a lower level of satisfaction from income rise as compared to individuals with a lower level of neuroticism. Therefore, it is possible that the nature of our personality trait particularly neuroticism determines whether we derive satisfaction in our life from income rise.

Findings of this study may have significant implications for various stakeholders interested into policies for abatement of poverty and increasing human well-being. Such policies should look into these complexities of human nature in judging the relationship between objective and subjective indicators of well-being. Particularly, the assumptions of universal parameters and referents (as most Economists believe) for human happiness and well-being can be erroneous and may not serve all. There can be substantial benefit in understanding how monetary incentives might influence people with different traits and life purposes.

### Limitations and Future Directions

Although this study included participants from the diverse socio-economic background, it is limited by sample size. Further, the results of this study are based on data collected from the samples residing in India. Specific cultural values of India (based on religious and spiritual orientations) and social structures based on caste systems might have influenced the results. Future studies should look into the phenomenon of satisfaction paradox to understand adaptation to life circumstances among people who report life satisfaction despite having very low income. There is a possibility of the existence of ‘learned helplessness’ among this group. It is possible that they have learned to accept their objectively deprived life circumstances after many unsuccessful attempts to change it for better. Whether people develop this referent for happiness as a result of learned helplessness or cultural values? This needs to be investigated in future research. The heterogeneity of conceptual referents and its role in explaining the relationship between income and life satisfaction should be further examined using larger data base. Further, research is needed to understand and clarify the division between “inner vs outer” orientated conceptual referents of happiness. The rationale behind such division is not very clear in some conceptual referents such as “virtue” and “satisfaction”. The roles of cultural values in developing conceptual referents can an interesting area of future research. Future studies should look into the role of other personality traits such as extraversion, conscientiousness in the relationship between income and life satisfaction.

## References

[r1] Argyle M. Lu L. (1990). The happiness of extraverts. Personality and Individual Differences, 11, 1011–1017. 10.1016/0191-8869(90)90128-E

[r2] Blanchflower D. G. Oswald A. J. (2004). Well-being over time in Britain and the USA. Journal of Public Economics, 88, 1359–1386. 10.1016/S0047-2727(02)00168-8

[r3] Boyce C. J. Wood A. M. (2011). Personality and the marginal utility of income: Personality interacts with increases in household income to determine life satisfaction. Journal of Economic Behavior & Organization, 78, 183–191. 10.1016/j.jebo.2011.01.004

[r4] Cheung F. Lucas R. E. (2014). Assessing the validity of single-item life satisfaction measures: Results from three large samples. Quality of Life Research, 23, 2809–2818. 10.1007/s11136-014-0726-4 24890827 PMC4221492

[r5] DeNeve K. M. Cooper H. (1998). The happy personality: A meta-analysis of 137 personality traits and subjective well-being. Psychological Bulletin, 124, 197–229. 10.1037/0033-2909.124.2.197 9747186

[r6] Diener E. (1984). Subjective well-being. Psychological Bulletin, 95, 542–575. 10.1037/0033-2909.95.3.542 6399758

[r7] Diener, E. (2009). Subjective well-being. In E. Diener (Ed.), *The science of well-being* (pp. 11-58). New York, NY, USA: Springer.

[r8] Diener, E., & Biswas-Diener, R. (2009). Will money increase subjective well-being? A literature review and guide to needed research. In E. Diener (Ed.), *The science of well-being* (pp. 119-154). New York, NY, USA: Springer.

[r9] Diener E. Diener M. Diener C. (1995). Factors predicting the subjective well-being of nations. Journal of Personality and Social Psychology, 69, 851–864. 10.1037/0022-3514.69.5.851 7473035

[r10] Diener, E., & Oishi, S. (2000). Money and happiness: Income and subjective well-being across nations. In E. Diener & E. M. Suh (Eds.), *Subjective well-being across cultures* (pp. 185-218). Cambridge, MA, USA: MIT Press.

[r11] Easterlin, R. A. (1974). Does economic growth improve the human lot? Some empirical evidence. In P. A. David & M. Reder (Eds.), *Nations and households in economic growth: Essays in honor of Moses (*pp. 89-125). New York, NY, USA: Academic Press.

[r12] Easterlin R. A. (1995). Will raising the incomes of all increase the happiness of all? Journal of Economic Behavior & Organization, 27, 35–47. 10.1016/0167-2681(95)00003-B

[r13] Howell R. T. Howell C. J. (2008). The relation of economic status to subjective well-being in developing countries: A meta-analysis. Psychological Bulletin, 134, 536–560. 10.1037/0033-2909.134.4.536 18605819

[r14] Inglehart, R. (1990). *Cultural shift in advanced industrial society*. Princeton, NJ, USA: Princeton University Press.

[r15] John, O. P., Donahue, E. M., & Kentle, R. L. (1991). *The Big Five Inventory – Versions 4a and 54*. Berkeley, CA, USA: University of California, Berkeley, Institute of Personality and Social Research.

[r16] Lakshmanasamy T. (2010). Are you satisfied with your income? The economics of happiness in India. Journal of Quantitative Economics, 8, 115–141.

[r17] Lucus, R. E., & Diener, E. (2009). Personality and subjective well-being. In E. Diener (Ed.), *The science of well-being* (pp. 75-102). New York, NY, USA: Springer.

[r18] Lykken D. Tellegen A. (1996). Happiness is a stochastic phenomenon. Psychological Science, 7, 186–189. 10.1111/j.1467-9280.1996.tb00355.x

[r19] Lyubomirsky S. (2001). Why are some people happier than others? The role of cognitive and motivational processes in well-being. The American Psychologist, 56, 239–249. 10.1037/0003-066X.56.3.239 11315250

[r20] Neff, D. (2009). *The satisfied poor: Evidence from south India* (BWPI Working Paper 7). Retrieved from http://hummedia.manchester.ac.uk/institutes/gdi/publications/workingpapers/bwpi/bwpi-wp-7109.pdf

[r21] Proto, E., & Rustichini, A. (2015). *Life satisfaction, income and personality* (IZA Discussion Paper No. 8837). Retrieved from http://ftp.iza.org/dp8837.pdf

[r22] Rojas M. (2005). A conceptual-referent theory of happiness: Heterogeneity and its consequences. Social Indicators Research, 74, 261–294. 10.1007/s11205-004-4643-8

[r23] Rojas, M. (2006). Well-being and the complexity of poverty: A subjective wellbeing approach. In M. McGillivray (Ed.), *Perspectives on human wellbeing* (pp. 182-206). Tokyo, Japan: United Nations University Press.

[r24] Rojas M. (2007). Heterogeneity in the relationship between income and happiness: A conceptual-referent-theory explanation. Journal of Economic Psychology, 28, 1–14. 10.1016/j.joep.2005.10.002

[r25] Rusting C. L. Larsen R. J. (1997). Extraversion, neuroticism, and susceptibility to positive and negative affect: A test of two theoretical models. Personality and Individual Differences, 22, 607–612. 10.1016/S0191-8869(96)00246-2

[r26] Sirgy M. J. Cole D. Kosenko R. Meadow H. L. Rahtz D. Cicic M. Nagpal N. (1995). A life satisfaction measure: Additional validational data for the Congruity Life Satisfaction measure. Social Indicators Research, 34, 237–259. 10.1007/BF01079198

[r27] Tatarkiewicz, W. (1976). *Analysis of happiness.* The Hague, The Netherlands: Martinus Nijhoff.

[r28] Veenhoven, R. (1984). *Conditions of happiness*. Dordrecht, The Netherlands: Kluwer Academic.

[r29] Vittarsø J. (2001). Personality traits and subjective well-being: Emotional stability, not extraversion, is probably the important predictor. Personality and Individual Differences, 31, 903–914. 10.1016/S0191-8869(00)00192-6

[r30] Zapf, W. (1984). Individuelle Wohlfahrt: Lebensbedingungen und wahrgenommene Lebensqualität. In W. Glatzer & W. Zapf (Eds.), *Lebensqualität in der Bundesrepublik Deutschland: Objektive Lebensbedingungen und subjektives Wohlbefinden* (pp. 13-26). Frankfurt, Germany: Campus.

